# Development of novel and green NiFe_2_O_4_/geopolymer nanocatalyst based on bentonite for synthesis of imidazole heterocycles by ultrasonic irradiations

**DOI:** 10.1038/s41598-020-68426-z

**Published:** 2020-07-15

**Authors:** Zoleikha Hajizadeh, Fateme Radinekiyan, Reza Eivazzadeh-keihan, Ali Maleki

**Affiliations:** Catalysts and Organic Synthesis Research Laboratory, Department of Chemistry, Iran University of Science and Technology, 16846-13114 Tehran, Iran

**Keywords:** Heterogeneous catalysis, Synthetic chemistry methodology, Magnetic properties and materials

## Abstract

Geopolymers as aluminosilicate inorganic polymers and eco-friendly building materials which can be used as substrate for different kinds of composite. In this research, according to the fabrication of geopolymer based on bentonite as a substrate and embedment of NiFe_2_O_4_ nanoparticles in the construction of this polymer, the synthesis of a new magnetic nanocomposite (NiFe_2_O_4_/geopolymer) was investigated for the first time. In order to describe its chemistry and morphology features, different analyses such as Fourier transform infrared spectroscopy, field-emission scanning electron microscopy and transmission electron microscopy images, Brunauer–Emmet–Teller adsorption–desorption isotherm, X-ray diffraction pattern, energy-dispersive X-ray analysis, thermogravimetric analysis, and vibrating-sample magnetometer analysis were used. The application of this novel nanocatalyst was studied for one-pot three-component condensation reaction of substituted imidazole derivatives by accelerated ultrasonic irradiations. Compared to the other conventional catalysts which were used for the synthesis of imidazole derivatives, the green synthesis method for fabrication of this heterogeneous and magnetic nanocatalyst, its high thermal stability, being eco-friendly, noticeable efficiency and easy reusability have become privileges to be superior.

## Introduction

Geopolymeric compounds as amorphous, three-dimensional alkali aluminosilicate binder materials were first introduced by Davidovits in 1978. In recent years, the perusal of geopolymeric materials has been increased due to their specific properties such as compliance with green chemistry, high mechanical performance as building materials, having micro or nano-porosity, thermal stability, high surface hardness, and acid and fire resistance^1^. These kind of materials can be fabricated by alkaline activation of various aluminosilicate materials including natural minerals such as kaolin^2^ and montmorillonite^3^, mineral resources like bentonite^4^, and also, industrial wastes such as fly ash^1, 5^. Among them, natural bentonite is one of the most multifunctional mineral clays with low cost, abundant accessibility, superb swelling capability, viscoelastic property at low concentration, superb absorption capacity and eco-friendly properties^1^. This phyllosilicate clay primarily consists of montmorillonite. Two main contributions to the surface charges of montmorillonite are the constant negative charges on the basal planes; which are related to the isomorphic substitutions and the pH-dependent charges developed on the hydroxyl groups at broken edges^6^. A localized charge distribution can be generated by a negative charge associated with a cation replacement (e.g., Al^+3^ for Si^+4^) in the tetrahedral sheet; while, a diffused negative charge can be achieved by the cation replacement (e.g., Mg^+2^ for Al^+3^) in the octahedral sheet^6^. This natural clay can be utilized as superb absorbent in various fields such as water purification and absorption of heavy metals^7^ due to its specific structure. The absorption of metal ions is accomplished by ion exchange process and as well as, the absorption of organic pollutants by interlayer space of bentonite^8, 9^. In the presence of strong alkaline solution, the rapid dissolution of aluminosilicate materials are conducted to form Si[OH]_4_ and Al[OH]_4_ oligomers. After the oligomers fabrication, the tetrahedral units are attached together to generate amorphous and three-dimensional geopolymer networks ([–(Si–O)_z_–Al–O–]_n_)^1, 10, 11^. According to the synthesis conditions, selection of raw materials, the ratio between silicon and aluminum, alkali element and other reinforcement options, the characteristics of geopolymers will be different and depend on these following items^12, 13^.

**Figure 1:**
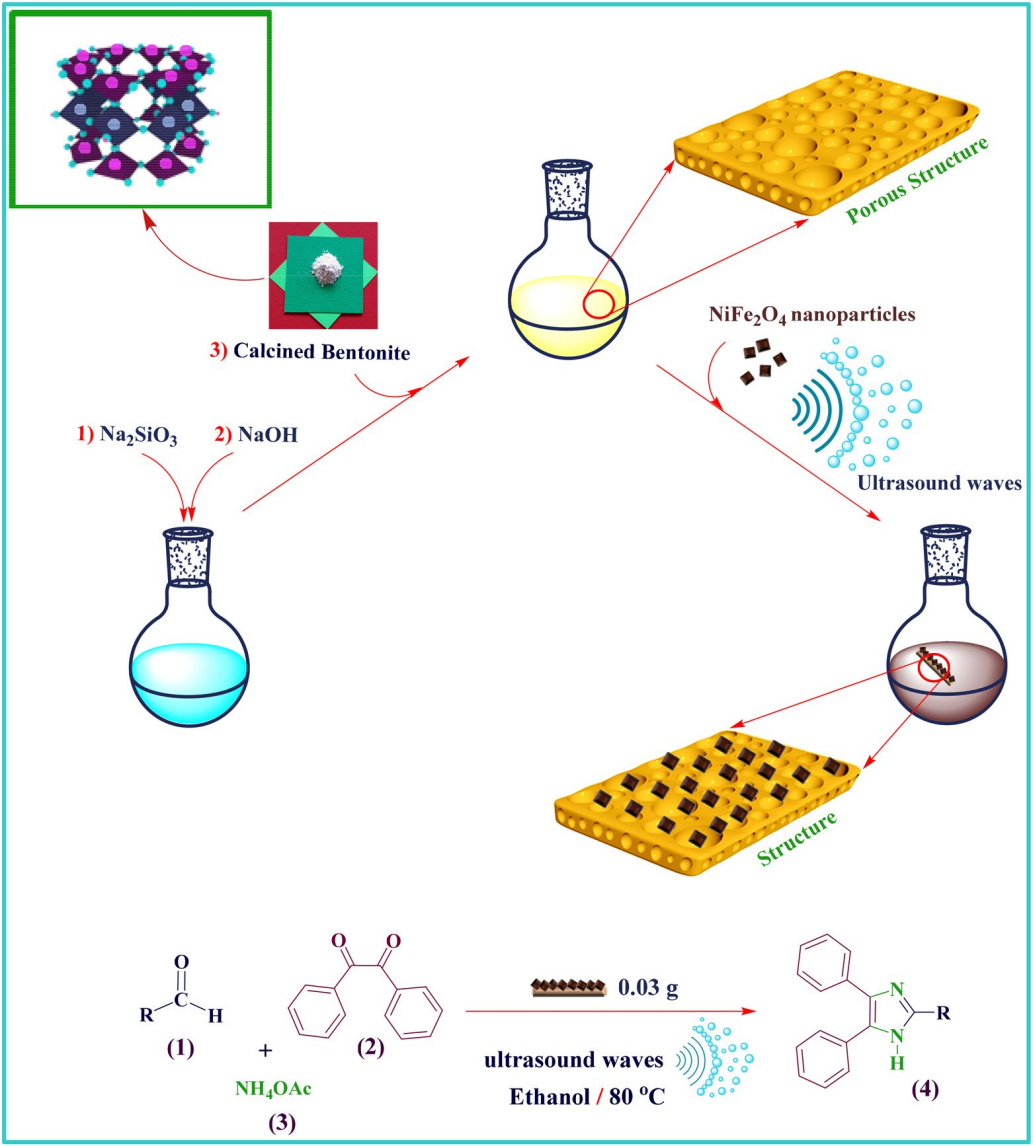
Synthesis of NiFe_2_O_4_/geopolymer nanocomposite and its catalytic application in three-component condensation reaction.

MFe_2_O_4_ (M = divalent metal ions, e.g. Ni, Co, Cu, Zn, Mg, Mn, Cd, etc.) as monocrystalline spinel ferrites have been generated a great deal of interest due to their particular characteristics such as magnetic, magneto resistive and magneto-optical properties^14^ and their extraordinary applications in industry^15^, medical science^16, 17^ and other scientific fields^18^,^19^. Nowadays, among different kinds of nanoparticle, NiFe_2_O_4_ nanoparticles as cubic ferromagnetic oxide with typical inverse spinel structure and exceptional magnetic and electromagnetic features^20^ have been attracted lots of attention. According to their applications, these nanoscale particles have been utilized in many fields such as absorbent^21^, catalysts^22^, biosensors^23^ and biomedicine^24, 25^ ; also, various techniques have been applied for the synthesis of nanoscale NiFe_2_O_4_ ferrites. These techniques consist of sol–gel^26^, co-precipitation^27^, mechanochemical^28^, micromulsion^29^ and sonochemical^30^. Sonochemistry based on ultrasonic irradiations is one of the most unique and green techniques for scientific and industrial investigations. In comparison to other conventional methods, its features such as controllability, accessibility, short time performance and accordance with green chemistry principles are distinctive; as well as, using ultrasonic irradiations was well appreciated by scientists in the preparation of new catalysts and running various organic reactions^31^. Acoustic cavitation theory can justify the rate increment of various reactions which are conducted by ultrasonic irradiations^31, 32^. According to this theory, the rate enhancement of synthetic reactions in few seconds is due to the formation of high microscopic pressures, the frequency of ultrasound, pulse duration, and as well, the nucleation of exposed medium^31, 33^. In accordance with green chemistry principles, one of the most substantial approaches to fabricate multifarious molecular compounds is multicomponent reactions^34^. Atomic-economy increment and improvement of resource and energy effectivity are carried out by multicomponent reactions; as well as, these kind of reactions have been created extensive pharmacological libraries due to their generation of fundamental compounds with valuable pharmaceutical properties^31^. Among these, multi-substituted imidazoles are well known as remarkable heterocyclic compounds due to their multifunctional capabilities and their various applications in biomedicine, pharmacology and other scientific fields. In many pharmaceutical compounds such as losartan, eprosartan^35^ and trifenagrel^36^, they act as effective backbones; also, their antitumor, anti-inflammatory, anti-allergic and analgesic activities have been proved^37–39^. In recent years, the synthesis of imidazole derivatives based on multicomponent reactions have been reported by wide range of catalysts including bulk scale catalysts like L-proline^40^ , DABCO^41^, Silica sulfuric acid^42^, and nanoscale catalysts such as ZnO nanorods^43^, nano MgO^44^. In comparison to bulk scale catalysts, nanocatalysts have been indicated unique privileges including large surface to volume ratio, enhancement in catalytic activity and efficient selectivity; however, their difficult separation from reaction has been restricted their applications^45, 46^. Therefore, the preparation and design of novel catalysts with two specific features including nanocatalytic activities and having magnetic properties can create better and efficient conditions for accomplishment of multicomponent reactions^44^. According to the development of novel methodologies based on green chemistry approaches, the synthesis, characterization and in situ preparation of our proposed new nanocomposite, NiFe_2_O_4_/geopolymer based on sonochemistry technique was propounded; the fabrication of this new catalyst was carried out in two steps; the first step included the preparation and synthesis of NiFe_2_O_4_ nanoparticles by coprecipitation method and the second step by intercalation method consisted of the in situ preparation of geopolymer based on bentonite and addition of NiFe_2_O_4_ spinel to the synthetic reaction of geopolymer^44^. Also, its application as a green, heterogeneous and recoverable nanocatalyst was investigated in the synthesis of substituted imidazole derivatives (**4a–p**) by three component condensation reaction of wide range of substituted aldehydes (**1**), benzil (**2**) and ammonium acetate (**3**) under the ultrasonic irradiations and green and nontoxic reaction condition (Fig. 1).

## Results and discussion

For the first time, the synthesis of NiFe_2_O_4_ nanocomposite based on geopolymer substrate and NiFe_2_O_4_ nanoparticles was introduced as a new magnetic nanocatalyst under the mild conditions. According to the Fig. 1, the fabrication of this new nanocatalyst was conducted in two synthesis steps; the first step included the preparation and synthesis of NiFe_2_O_4_ nanoparticles by coprecipitation method and the second step by intercalation method consisted of the in situ preparation of geopolymer based on bentonite clay and the addition of NiFe_2_O_4_ spinels to the synthetic solution of geopolymer. The formation of hydrogen bonds between hydroxyl groups of geopolymer and hydroxyl groups of synthetic NiFe_2_O_4_ nanoparticles were conducted and then, the synthetic magnetic nanoparticles were intercalated into the porous structure of geopolymer. Various detection techniques including Fourier-transform infrared (FT-IR) spectroscopy for characterizing the functional groups, energy dispersive X-ray (EDX) analysis to identify the elemental composition, using field-emission scanning electron microscopy (FE-SEM) and transmission electron microscopy (TEM) images for observing the morphology and size of the NiFe_2_O_4_/geopolymer nanocomposite, Brunauer–Emmet–Teller (BET) adsorption–desorption isotherm to indicate the surface area, pore volume and pore size of geopolymer and NiFe_2_O_4_/geopolymer nanocomposite, X-ray diffraction (XRD) pattern for determining the crystalline phase of bentonite clay, synthetic geopolymer and NiFe_2_O_4_/geopolymer nanocomposite, thermogravimetric (TG) analysis for the evaluation of thermal behavior and stability and vibrating-sample magnetometer (VSM) analysis for the characterization of magnetic properties of synthetic NiFe_2_O_4_/geopolymer nanocomposite are discussed respectively.

### Characterization of the NiFe_2_O_4_/geopolymer nanocomposite

#### FT-IR analysis

**Figure 2:**
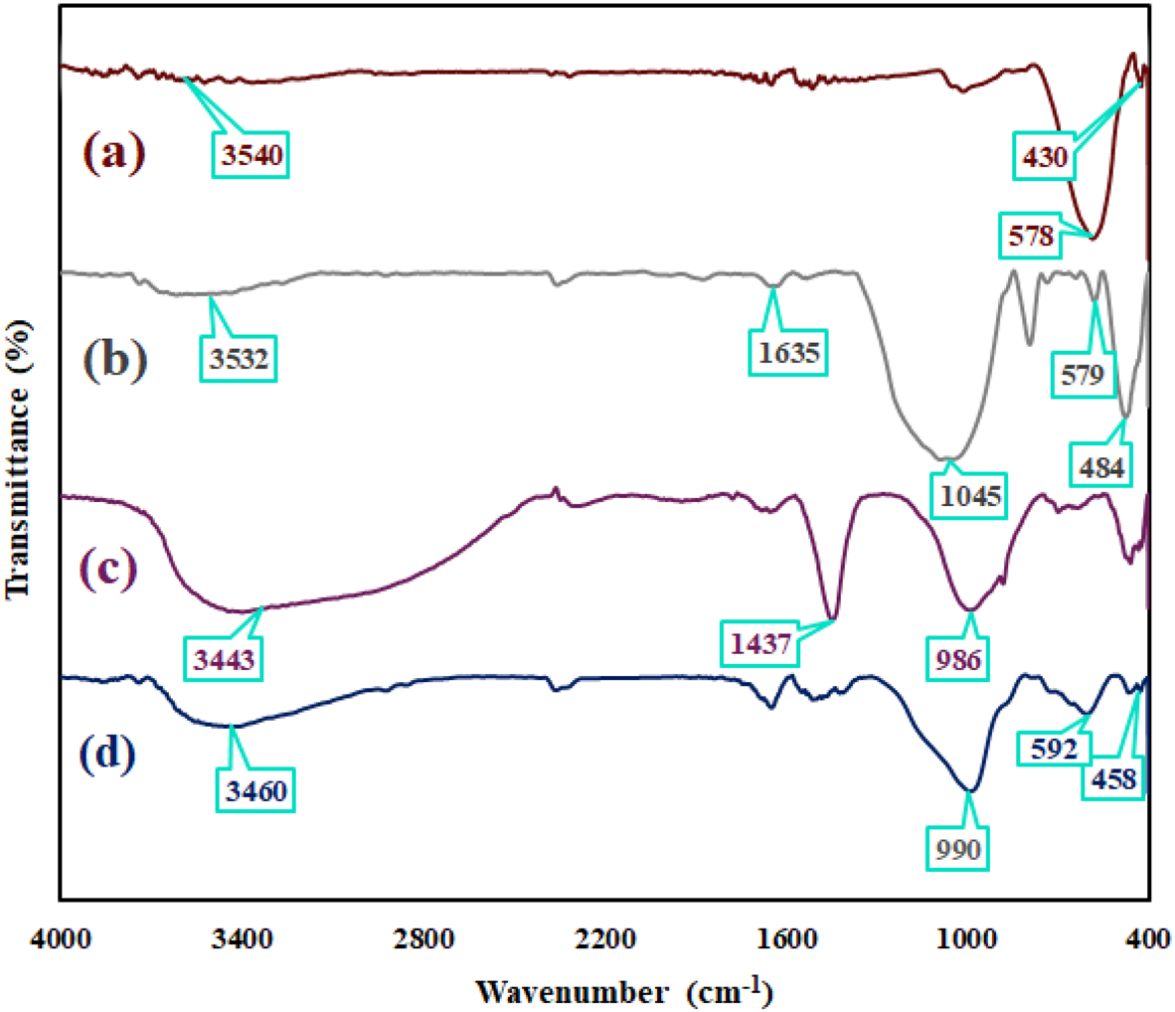
FT-IR spectra of (**a**) NiFe_2_O_4_ nanoparticles, (**b**) bentonite, (**c**) geopolymer and (**d**) NiFe_2_O_4_/geopolymer nanocomposite.

As could be seen in Fig. 2, the formation of the NiFe_2_O_4_/geopolymer nanocomposite was confirmed by FT-IR spectroscopy technique. Figure 2a illustrated the FT-IR spectrum of NiFe_2_O_4_ nanoparticles. The two absorption bands of octahedral complex around 427 cm^−1^ and tetrahedral complex around 587 cm^−1^ were observed in the spectrum^47^. Figure 2b indicated the FT-IR spectrum of bentonite clay. The absorption band around 3,543 cm^−1^ was indicated for stretching vibration mode of OH groups. Also, an overtone band which is related to the bending vibration of water was observed around 1637 cm^−1^ ^48^. The strong absorption band around 1,046 cm^−1^ was considered as Si–O stretching vibration mode^49^. The bands around 578 cm^−1^ and 475 cm^−1^ were indicated the Al–O–Si and Si–O–Si bending vibration modes respectively^50^. Figure 2c as a FT-IR spectrum of geopolymer, has conceded the fabrication of polymer based on bentonite clay. According to this spectrum, the band amplification and high transmittance percentage of OH groups have been increased; also, an observed strong absorbance band around 1,430 cm^−1^ ^51, 52^ was confirmed the existence of C–O–C asymmetric vibration mode of CO_3_^2–^ ion which has been determined in all spectra of geopolymer samples. A broad band at range of 900–1,000 cm^−1^ (986 cm^−1^) was attributed to the Si–O–Si stretching vibration mode^53^. Besides, due to the formation of geopolymer structure, the Si–O–Si stretching vibration mode has shifted to the lower wavenumbers^53^. A broad band Fig. 2d illustrated the FT-IR spectrum of synthetic nanocomposite. According to the FT-IR spectrum of synthetic nanocomposite, the strong Si–O–Si stretching vibration mode (990 cm^−1^) was assigned in the related spectrum (Fig. 2d). A considerable reduction in amplification of O–H stretching band which was observed is due to the interaction between magnetic NiFe_2_O_4_ nanoparticles and OH groups of geopolymer. As mentioned before, two absorbance bands at 578 cm^−1^ and 475 cm^−1^ were characterized as bending vibration modes of Al–O–Si and Si–O–Si. Apart from these mentioned bands, as well, the absorption bands of octahedral and tetrahedral complexes of NiFe_2_O_4_ nanoparticles were also determined at 427 cm^−1^ and 587 cm^−1^. Conforming to these observations, it can be concluded that the absorbance bands of NiFe_2_O_4_ nanoparticles have overlapped with the mentioned absorption bands of synthetic geopolymer.

#### EDX analysis

**Figure 3:**
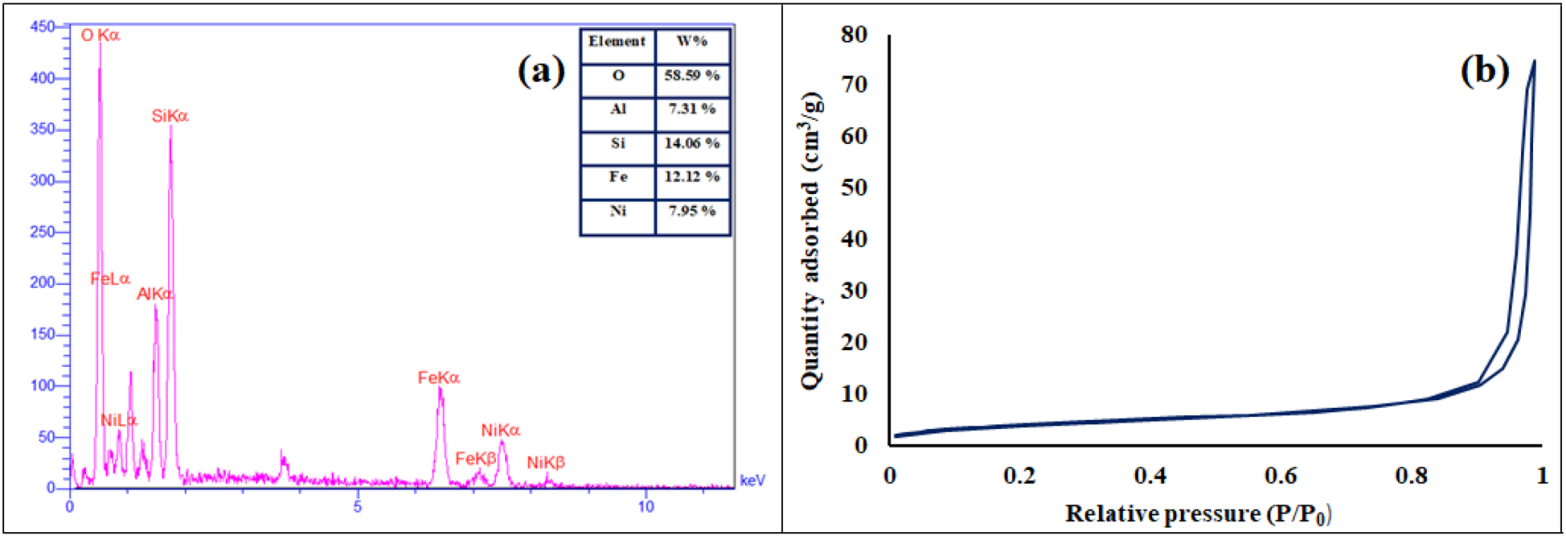
(**a**) EDX spectrum of NiFe_2_O_4_/geopolymer nanocomposite, (**b**) nitrogen adsorption tubular isotherm of NiFe_2_O_4_/geopolymer nanocomposite.

EDX analysis as a useful and efficient method can be utilized for elemental composition of different kinds of materials. The EDX analysis of NiFe_2_O_4_/geopolymer nanocomposite was indicated in Fig. 3a. Based on the obtained results from EDX spectrum of designed NiFe_2_O_4_/geopolymer nanocomposite, the presence of iron, nickel and oxygen peaks was related to the structure of synthetic magnetic NiFe_2_O_4_ nanoparticles. As well as, alongside of oxygen peak, the aluminum and silicon peaks were attributed to the fabricated and inorganic substrate, geopolymer.

#### Contexture characterization of geopolymer and NiFe_2_O_4_/geopolymer nanocomposite

The BET analysis of geopolymer and NiFe_2_O_4_/geopolymer nanocomposite was evaluated by nitrogen gas adsorption. As could be seen, the nitrogen adsorption tubular isotherm of NiFe_2_O_4_/geopolymer nanocomposite is indicated in (Fig. 3b). According to the obtained results (Table was attached in supplementary information file), the determination of pore sizes of geopolymer (14.28 nm) and NiFe_2_O_4_/geopolymer nanocomposite (19.82 nm), it could be concluded that the substrate and magnetic nanocomposite were categorized as porous materials^54^, as well as, in comparison to geopolymer, the surface decrement and volume increment of pores in the NiFe_2_O_4_/geopolymer nanocomposite were related to the in situ preparation of synthetic nanocomposite and addition of NiFe_2_O_4_ nanoparticles during the geopolymer lattice formation and generation of polymeric lattice with bigger sizes and volumes.

#### FE-SEM and TEM imaging

**Figure 4:**
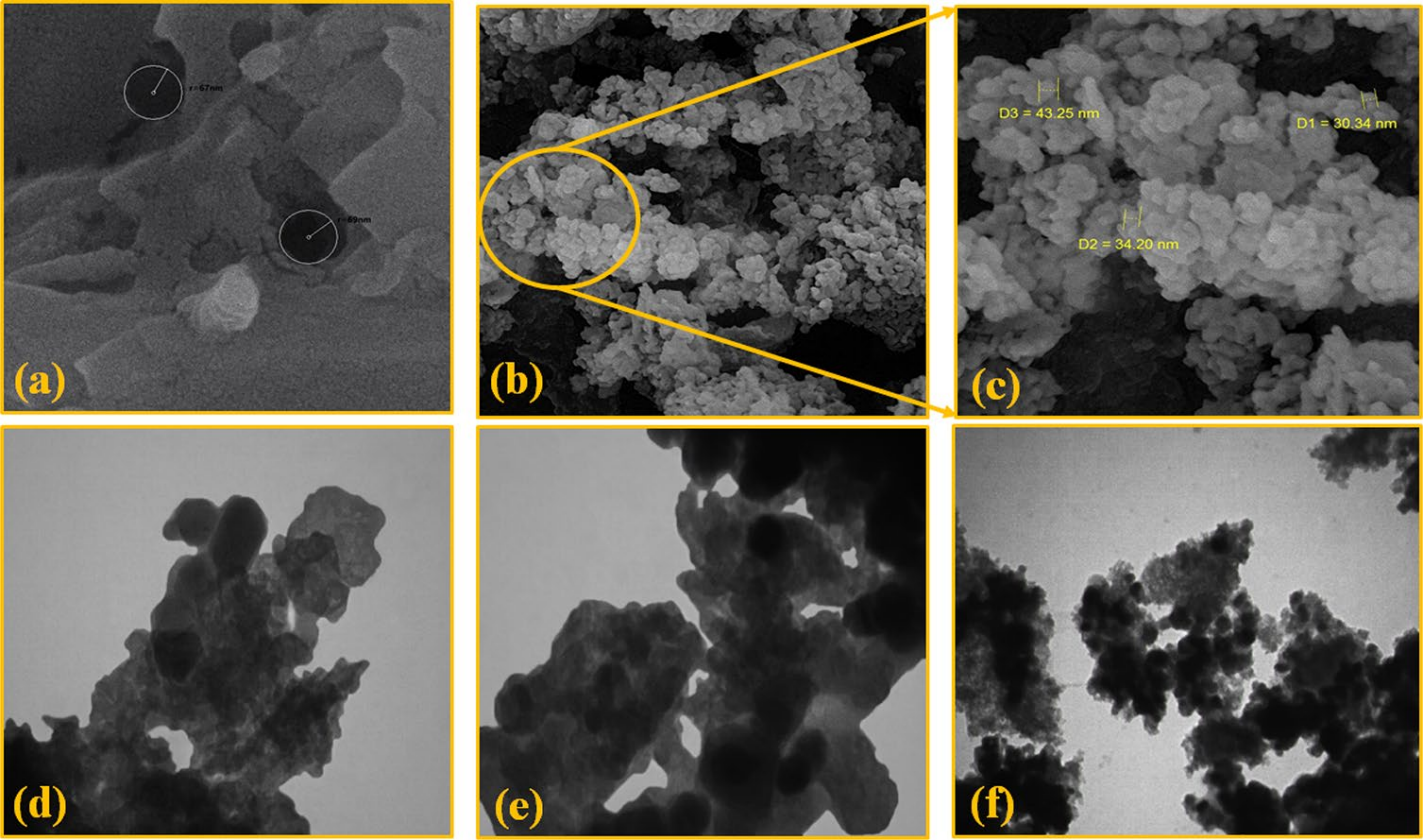
FE-SEM images of (**a**) geopolymer, (**b**,**c**) magnetic NiFe_2_O_4_/geopolymer nanocomposite, (**d**,**e**,**f**) TEM images of magnetic NiFe_2_O_4_/geopolymer nanocomposite.

The FE-SEM and TEM images of geopolymer and magnetic NiFe_2_O_4_/geopolymer nanocomposite were indicated in Fig. 4a–f. Given the obtained results from the surface imaging, the pores of geopolymer were well characterized with an average diameter between 130 to 140 nm (Fig. 4a). After the addition of NiFe_2_O_4_ nanoparticles and preparation of NiFe_2_O_4_/geopolymer nanocomposite, the FE-SEM images from the external surfaces showed that the pores of geopolymer substrate had filled; also, nickel ferrite nanoparticles on the surface of the substrate and inside their pores were well observed. The cubic shape was considered for NiFe_2_O_4_ nanoparticles with almost uniform nanoscale size; as well as, their average diameter (30.34 ± 10 nm) was well determined (Fig. 4b–c). Furthermore, the presence and distribution of NiFe_2_O_4_ nanoparticles were characterized by the TEM images of NiFe_2_O_4_/geopolymer nanocomposite (Fig. 4d–f).

#### XRD pattern

**Figure 5:**
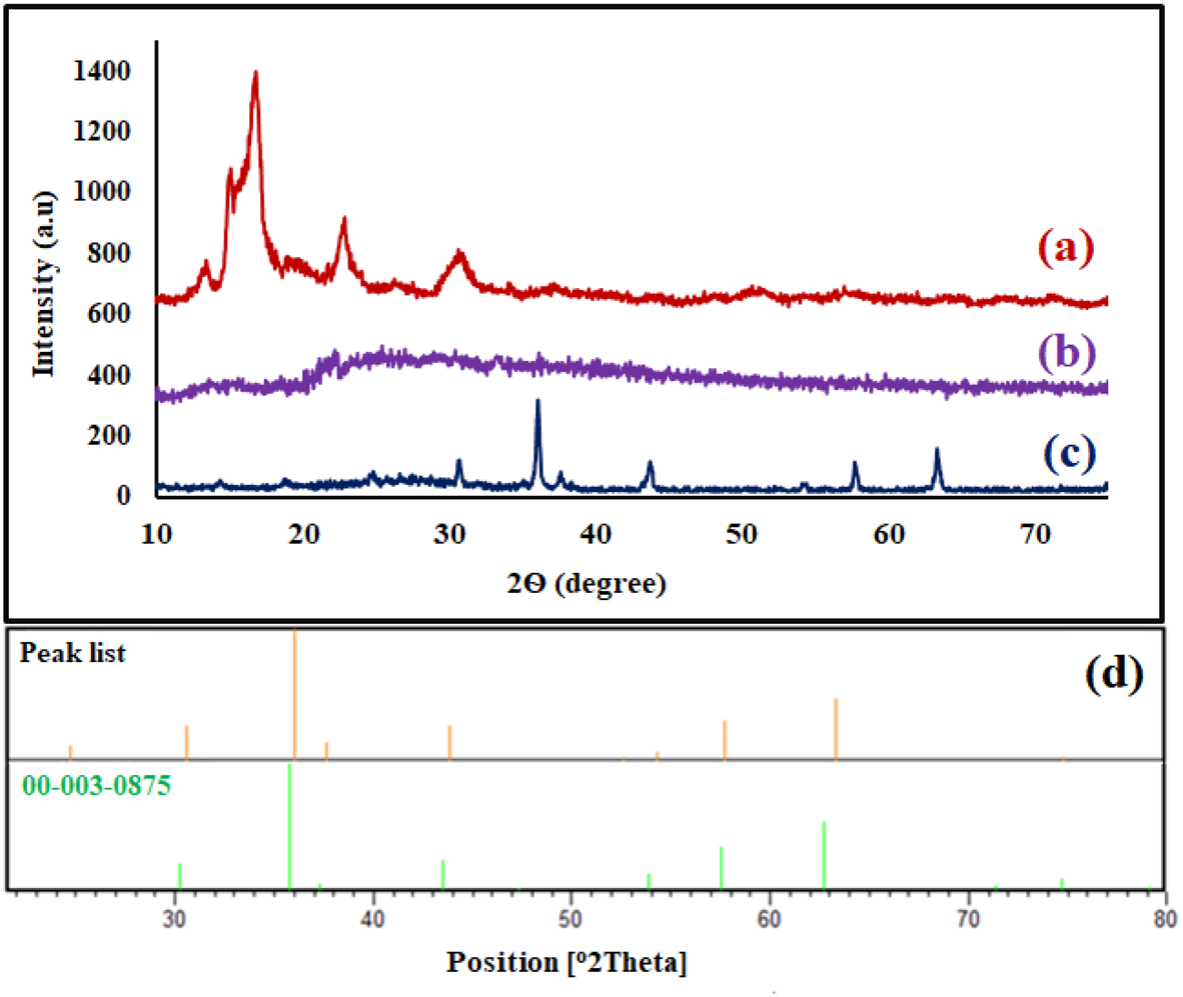
XRD pattern of (**a**) bentonite, (**b**) geopolymer, (**c**) NiFe_2_O_4_/geopolymer nanocomposite, (**d**) references of cubic NiFe_2_O_4_ nanoparticles.

As could be seen, the XRD patterns of bentonite, geopolymer, and NiFe_2_O_4_/geoplymer are indicated in Fig. 5a–c. Taking into account, the synthetic process of designed magnetic nanocomposite, the crystalline peaks of bentonite with diffraction angles (2θ) (18.33, 19.97, 21.64, 27.66, 35.60, 69.81) (Fig. 5a) have eliminated in the XRD pattern of designed nanocomposite. The amorphous character of geoplymer was appeared at the diffraction angle range of 2θ = 20–35^55^ (Fig. 5b). Apart from the amorphous peak, other mentioned diffraction angles (2θ) (30.61, 36.03, 44.31, 54.25, 57.70, 74.77) in XRD pattern (Fig. 5c) are complied with the JCPDS card number of cubic NiFe_2_O_4_ nanoparticles (JCPDS card No. 00-003-0875); therefore, the presence of NiFe_2_O_4_ nanoparticles were confirmed in the structure of synthetic NiFe_2_O_4_/geopolymer nanocomposite (Fig. 5d).

#### Thermogravimetric analysis

**Figure 6:**
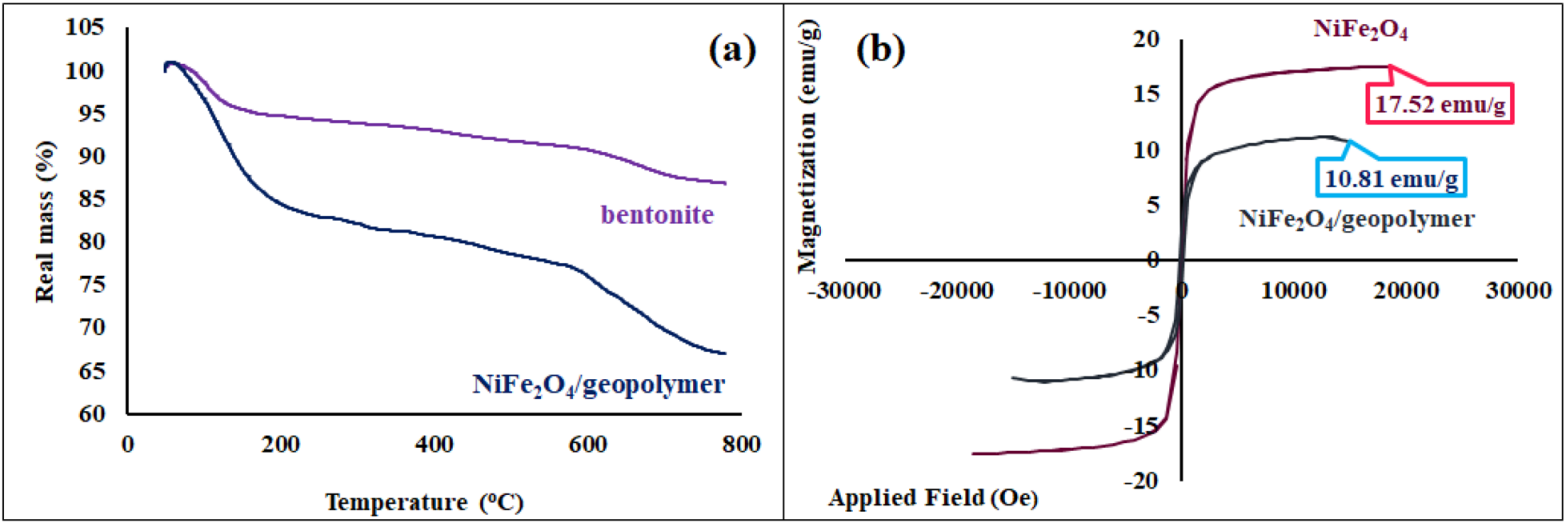
(**a**) TGA curves of the bentonite clay and NiFe_2_O_4_/geopolymer nanocomposite, (**b**) hysteresis loop curve of NiFe_2_O_4_ nanoparticles and nanocomposite.

To evaluate the thermal behavior and stability of NiFe_2_O_4_/geopolymer nanocomposite, the thermogravimetric analysis was conducted at the constant thermal rate of 10 °C/min in the air atmosphere. As can be seen, the decomposition result of bentonite clay and the NiFe_2_O_4_/geopolymer nanocomposite are observed in Fig. 6a. In comparison of bentonite clay, two mass reductions of NiFe_2_O_4_/geopolymer nanocomposite were more considerable. As illustrated, the first mass reduction of NiFe_2_O_4_/geopolymer nanocomposite at the temperature range of 50–250 °C was related to the elimination of water and feasible impurities. In comparison to bentonite and NiFe_2_O_4_/geopolymer nanocomposite, the losing weight for magnetic nanocomposite in the range of 50–250 °C was almost 15% more than bentonite clay. This result confirmed the polymeric structure of magnetic nanocomposite, and its substantial porosity have prepared more absorption condition for water and excessive impurities. The second mass reduction of NiFe_2_O_4_/geopolymer nanocomposite at the temperature range of 450–800 °C was attributed to the destruction of carbonate^56^ which existed in the structure of synthetic magnetic nanocomposite.

#### VSM analysis

Magnetic saturation value of functionalized and unfunctionalized magnetic nanostructures can be determined by VSM analysis. The hysteresis loop curves of the bare magnetic NiFe_2_O_4_ nanoparticles and NiFe_2_O_4_/geopolymer nanocomposite are shown in Fig. 6b. As could be seen, the saturation magnetization value of NiFe_2_O_4_/geopolymer nanocomposite was reduced compared to bare NiFe_2_O_4_ nanoparticles. The loading of NiFe_2_O_4_ nanoparticles on the geopolymer led to a decrease in the saturation magnetization value of them. Therefore, the synthetic geopolymer with porous structure and abundant hydroxyl groups could be applied as a good host and substrates for magnetic nanoparticles. However, NiFe_2_O_4_/geopolymer nanocomposite showed the acceptable magnetic property.

### Investigation of Catalytic activity of the NiFe_2_O_4_/geopolymer nanocomposite in synthesis of imidazole derivatives

#### Optimization of different parameters

In order to acquire the optimum parameters for the synthesis of imidazole derivatives, the one pot three-component condensation reaction of benzil (0.8 mmol), benzaldehyde (0.8 mmol) and ammonium acetate (2.0 mmol) were examined by various conditions (Table S2, entries 1–17). As can be seen the catalytic activity of synthetic nanocomposite was evaluated by three kinds of method including room temperature, reflux and ultrasonic condition (Table S2, entries 1–5). It was noteworthy that the best result was observed by ultrasonic bath and using of 0.03 g nanocatalyst (Table S2, entry 5). After the method determination, the best type of solvent for the reaction was analyzed. Based on the obtained results, the highest yield percentage was observed in ethanol, the green and non-toxic solvent (Table S2, entry 5). In a close and precise investigation, the catalytic activity of bentonite, synthetic substrate (geopolymer) and NiFe_2_O_4_ magnetic nanoparticles was evaluated before the value optimization of catalyst (Table S2, entries 11–13). In comparison to bentonite which did not show any catalytic activity, the activity of geopolymer was 20%, also, the activity of NiFe_2_O_4_ nanoparticles was reported 38%. By combination of geopolymer and NiFe_2_O_4_ nanoparticles and introducing a new magnetic nanocomposite, the catalytic activity of synthetic nanocomposite was increased up to 65%. The loading of NiFe_2_O_4_ nanoparticles in the surface of geopolymer and the existed hydroxyl groups in the structure of geopolymer caused to increase the efficiency of the nanoparticles by the distribution of magnetic NiFe_2_O_4_ nanoparticles and preventing the aggregation of the nanoparticles. Also, their activated hydroxyl groups in geopolymer have enhanced the catalytic activity. In other words, the synergic effect of hydroxyl groups of geopolymer and NiFe_2_O_4_ in the NiFe_2_O_4_/geopolymer increased the catalytic activity. Apart from method selection and type of solvent, the amount of magnetic catalyst was optimized (Table S2, entries 13–17). Generalization of optimized reaction condition and desirable performance of the NiFe_2_O_4_/geopolymer nanocatalyst were examined by the wide range of substituted aldehydes. A variety of substituted imidazole compounds (**4a–p**) were fabricated by 0.03 g of NiFe_2_O_4_/geopolymer nanocatalyst under the ultrasonic irradiations in the short reaction time. The corresponding results are represented in Table 1.

**Table 1:** Synthesis of 2,4,5-trisubstituted imidazole derivatives by using NiFe_2_O_4_/geopolymer nanocatalyst.

Entry	R	Product	Time (min)	Yield^a^ (%)	Melting point (°C) Observed	Melting point (°C) Reported
1	Benzaldehyde	**4a**	18	91	277–279	278–280^57^
2	4-Chlorobenzaldehyde	**4b**	18	88	262–265	262–264^57^
3	2-Chlorobenzaldehyde	**4c**	25	84	196–198	196–198^35^
4	3-Chlorobenzaldehyde	**4d**	26	81	296–298	296–297^58^
5	2,4-Dichlorobenzaldehyde	**4e**	28	89	174–177	176–178^35^
6	2,6-Dichlorobenzaldehyde	**4f**	33	84	236–238	236–238^59^
7	4-Bromobenzaldehyde	**4 g**	27	87	265–267	264–266^60^
8	4-Methoxybenzaldehyde	**4 h**	30	86	232–234	233–234^61^
9	3,4,5-Trimethoxybenzaldehyde	**4i**	33	82	259–261	261–262^62^
10	2-Methoxybenzaldehyde	**4j**	35	85	210–212	209–211^63^
11	4-Methylbenzaldehyde	**4 k**	33	88	231–233	230–232^64^
12	4-Hydroxybenzaldehyde	**4 l**	38	87	261–263	260–262^65^
13	2-Nitrobenzaldehyde	**4 m**	30	86	231–233	230–232^63^
14	4-Nitrobenzaldehyde	**4n**	26	84	234–236	235–238^66^
15	4-Fluorobenzaldehyde	**4o**	32	92	248–250	247–249^67^
16	Furfural	**4p**	29	90	237–239	237–239^68^

^a^Isolated yield.

#### Mechanism study of the NiFe2O_4_/geopolymer nanocatalyst in synthesis process of imidazole derivatives

**Figure 7:**
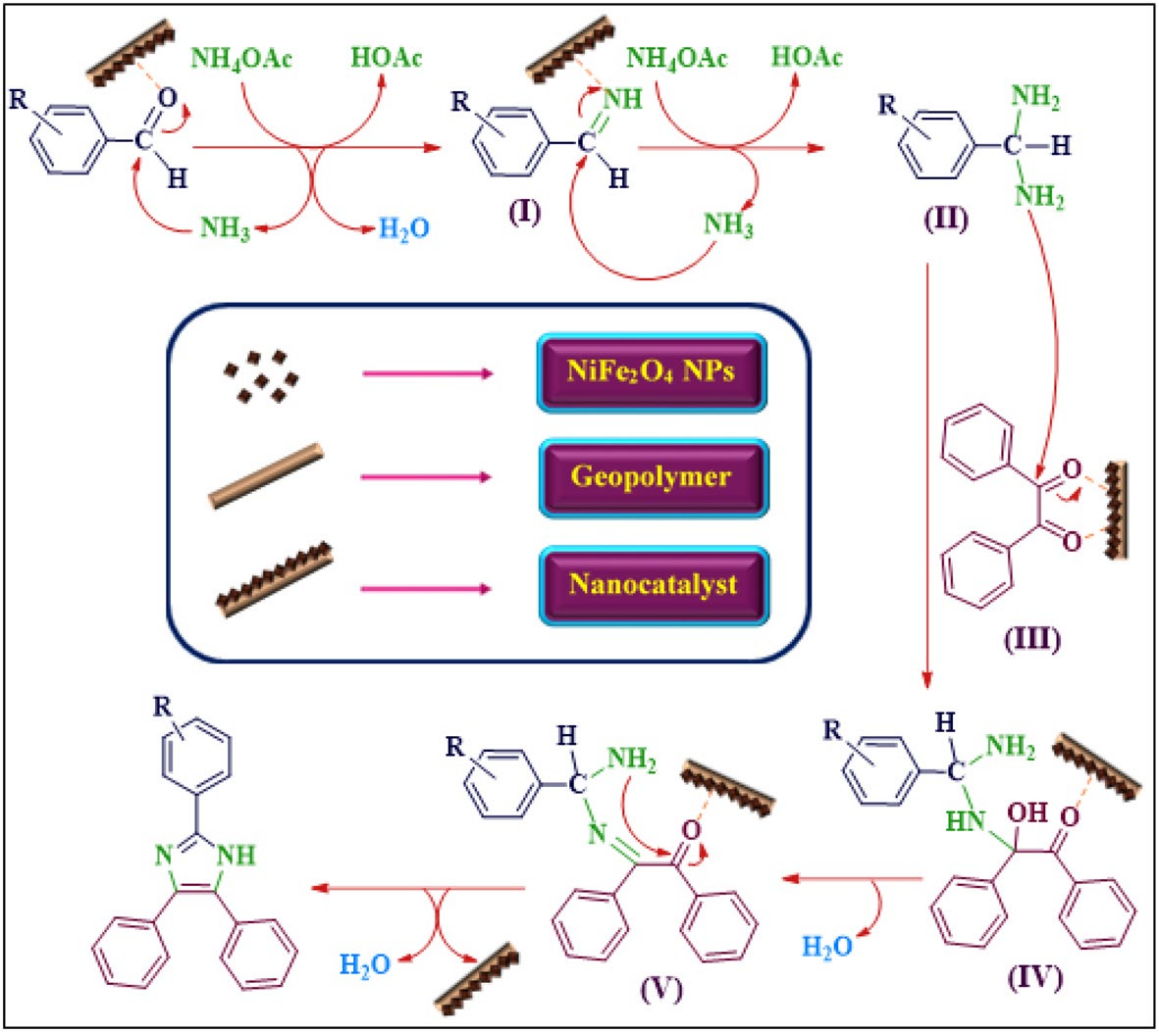
The proposed mechanism for synthesis of imidazole derivatives and the performance of magnetic nanocatalyst in the reaction.

The outline of the proposed mechanism for synthesis of imidazole derivatives was indicated in Fig. 7. According to the previous studies on applying metal oxide and ferrite nanoparticles as Lewis acid catalyst^44, 69^, first of all, the carbonyl group was activated by Lewis acid sites of magnetic nanocatalyst. Activated aldehyde was attacked by NH_3_ nucleophile. Then, the dehydration process was conducted in order to form imine intermediate (**I**) forming diamine intermediate (**II**) by nucleophilic attack of another NH_3_ and its nucleophilic attack to activated carbonyl groups of benzil (**III**) generated intermediate (**IV**). Intermediate (**V**) was formed by release of water and the cyclization process was carried out by intermolecular nucleophilic attack. Eventually, the 2,4,5-trisubstituted imidazole product (**VI**) was fabricated by dehydration process and released of catalyst. After the reaction completion, the magnetic nanocatalyst was separated by the external magnet from the reaction ambient and could be utilized for several runs.

#### Evaluation of catalyst reusability for several runs

One of the most important aspect of a catalyst is its recovery and reusability. For this purpose, first, the NiFe_2_O_4_/geopolymer nanocatalyst was recovered and separated from the reaction ambient by an external magnet. Then, the nanocatalyst washed with ethanol and dried at 70 °C for an overnight. After the separation process, the nanocatalyst was reused with the same amount in the model reaction. The recycle-ability of synthetic nanocatalyst was substantial and after 8 runs (Fig. S1), no significant reduction was observed in its catalytic activity. In addition, the stability of recycled nanocatalyst was confirmed by the FT-IR and EDX analyses (Fig. S2 and S3).

## Experimental

### General

In this study, the bentonite nanoclay (Bentonite Aldrich 682659) was applied as a primary source for the synthesis of a geopolymer. Also, all chemicals such as nickel nitrate, iron nitrate (III), sodium hydroxide, sodium silicate, and other solvents and reagents were analytical grade and purchased from Merck and Aldrich company. Melting points were measured on an Electrothermal 9,100 apparatus and are uncorrected. FT-IR spectra were recorded on a Shimadzu IR-470 spectrometer by the method of KBr pellets. ^1^H NMR and ^13^C NMR spectra were recorded with a Bruker DRX-500 Avance spectrometer at 500 and 125 MHz, respectively. FE-SEM images were taken with KYKY-EM3200. TEM images were prepared by ZEISS device (EM10C-100 kV model, Germany) and the physical absorption of gas molecules on the surface area of geopolymer and the synthetic magnetic NiFe_2_O_4_/geopolymer nanocomposite were carried out by BET technique (micromeritics ASAP 2020). EDX analysis was recorded with a Numerix DXP–X10P. XRD patterns of the solid powders were carried out using a JEOL JDX–8030 (30 kV, 20 mA). TG analysis was taken by Bahr-STA 504 instrument under the air atmosphere, VSM analysis was carried out by LBKFB model-magnetic kavir, as well as, the fabrication and catalytic application of synthetic nanocomposite was evaluated by Elmasonic device, S model (60 H). The identification of products was accomplished by comparison of their spectroscopic and analytical data with those of authentic samples.

### Preparation of NiFe_2_O_4_ nanoparticles

According to the previous reports about the fabrication of NiFe_2_O_4_ nanoparticles by coprecipitation method, the preparation of NiFe_2_O_4_ nanoparticles was reported by these following steps: firstly, 400 mL of nickel nitrate hexahydrate solution (Ni(NO_3_)_2_. 6 H_2_O) with the molarity of 0.1 M and 400 mL of iron nitrate (III) nonahydrate (Fe(NO_3_)_3_. 9H_2_O) with the molarity of 0.2 M were prepared separately. After solutions preparation, the two solutions were mixed together and stirred for 20 min at room temperature and then followed by continuous heating up to 90 °C. After a while, the addition of preheated 400 mL of NaOH solution with molarity of 3 M to the mixture was conducted quickly. The solution was heated for 1 h at 90 °C and then cooled down to room temperature. After the cooling process, in order to achieve pH value of 7, the solution was washed with distilled water. Afterwards, it was dried in the oven at 100 °C overnight. Finally, in order to have pure and well distinct NiFe_2_O_4_ spinel without any excessive impurity, the calcination process of powder was carried out for 6 h at 800 °C.

### In situ preparation of NiFe_2_O_4_/geopolymer nanocomposite by ultrasonic irradiation

The preparation of NiFe_2_O_4_/geopolymer nanocomposite based on bentonite was accomplished by using ultrasonic technique. In order to fabricate geopolymer^4^, first, 2.00 g of NaOH powder was added to 1.40 g of Na_2_SiO_3_ powder. In this synthetic process, the ratio of NaOH /Na_2_SiO_3_, SiO_2_/Al_2_O_3_ were 1.4 and 2, respectively. Then, the addition of 50 mL of distilled water was carried out to the mixture of the powders. After the dissolution of powders, the solution was left for ten min. Then, 1.20 g of calcined bentonite powder (500 °C for 6 h) was added to the solution and stirred at room temperature. Due to the addition of bentonite, a solution which called slurry was constructed. After its formation, 0.04 g of well distinct NiFe_2_O_4_ spinel was added to the slurry solution and dispersed by an ultrasonic bath for 45 min at room temperature. Finally, the resulted nanocomposite was obtained by setting the solution into the oven for 72 h at 70 °C.

### General procedure for the synthesis of 2,4,5-trisubstituted imidazole derivatives (4a–q)

Taking into account the ultrasonic condition, the reaction of a mixture consists of benzil (0.8 mmol), several types of substituted aldehyde (0.8 mmol), ammonium acetate (2.0 mmol) and NiFe_2_O_4_/geopolymer nanocomposite as catalyst (0.03 g) was carried out in the ethanol as a green solvent. The progression of the reaction was monitored by TLC in appropriate times. According to the reaction accomplishment, the separation of catalyst was conducted by an external magnet, also, 2,4,5-trisubstituted imidazole derivatives were attained by recrystallization process in ethanol. The synthesis of All the known product were approved by the comparison of their melting points with those of authentic literature samples (Table 1) and in some case the ^1^H NMR and ^13^C NMR spectra were taken (supplementary information file).

### Spectral data of selected products

2,4,5-triphenyl-1*H*-imidazole (**4a**): ^1^H NMR (500 MHz, DMSO): δ_H_ (ppm) = 7.21–7.56 (13H, m, H-Ar), 8.08–8.09 (2H, d, J = 7.8, H-Ar), 12.71 (1H, s, NH); ^13^C NMR (125 MHz, DMSO); δ_C_ (ppm) = 125.1, 128.1, 128.6, 130.3, 145.4.

4,5-diphenyl-2-(3,4,5-trimethoxyphenyl)-1*H*-imidazole (**4i**): ^1^H NMR (500 MHz, DMSO): δ_H_ (ppm) = 3.71 (3H, s, H-OMe), 3.87 (6H, s, H-OMe), 7.22–7.55 (13H, m, H-Ar), 12.65 (1H, s, NH); ^13^C NMR (125 MHz, DMSO); δ_C_ (ppm) = 55.97, 60.07, 102.62, 125,83, 137.72, 145,39, 153.10.

## Conclusions

In summary, according to the substantial aspects of sonochemistry, using this technique can be leaded to generate catalysts with high efficiency. In this work, a new magnetic nanocomposite according to geopolymer and NiFe_2_O_4_ nanoparticles was fabricated under the ultrasonic condition. The characterization and features of NiFe_2_O_4_/geopolymer as a new magnetic nanocomposite were carried out by wide range of spectroscopic techniques such as FT-IR, EDX, FE-SEM, TEM, BET, XRD, TGA, VSM analysis. According to the obtained results, NiFe_2_O_4_/geopolymer nanocomposite was categorized as mesoporous compounds. The volume and size of pores of synthetic nanocomposite were increased due to the presence and addition of magnetic and cubic NiFe_2_O_4_ nanoparticles during the in situ preparation of geopolymer; as well as, their presence has induced a magnetic property to this synthetic nanocomposite. Its thermal stability was substantial due to the presence of polymeric structure of geopolymer. The catalytic activity of NiFe_2_O_4_/geopolymer nanocomposite was evaluated by one pot three condensation reaction of 2,4,5-triaryl-1*H*-imidazoles. According to the time of reaction and considerable isolated organic products, this new nanocomposite demonstrated fundamental catalytic activity. In spite of reaction accomplishment by ultrasonic irradiations, the chemically and mechanically stability of proposed nanocatalyst was considerable and its reusability was determined by various analyses including FT-IR and EDX.

## Supporting information

Additional supporting information including the table of BET analysis, ^1^H and ^13^C NMR of the products, the table of optimizing of the reaction conditions, spectroscopic characterization data of the FT-IR spectrum and the EDX analysis of recycled nanocatalyst and diagram of reusability of nanocatalyst can be found in the online version of this article at the publisher’s web site.

## Supplementary information

Supplementary file1 (PDF 856 kb)
