# Supplementary material for: Development of novel and green NiFe2O4/geopolymer nanocatalyst based on bentonite for synthesis of imidazole heterocycles by ultrasonic irradiations
Source: Sci Rep. 2020 Jul 15;10:11671. doi: 10.1038/s41598-020-68426-z (PMC7363903; doi:10.1038/s41598-020-68426-z)
Supplement: Supplementary file 1 — Supplementary file1 (PDF 856 kb) [file 41598_2020_68426_MOESM1_ESM.pdf]

Supplementary information

Development of novel and green $NiFe_2O_4$/ geopolymer nanocatalyst based on bentonite for synthesis of imidazole heterocycles by ultrasonic irradiations

Zoleikha Hajizadeh, Fateme Radinekiyan,[1] Reza Eivazzadeh-keihan, Ali Maleki*

Catalysts and Organic Synthesis Research Laboratory, Department of Chemistry, Iran University of Science and Technology, Tehran 16846-13114, Iran

**Corresponding author. E-mail: maleki@iust.ac.ir*

[1] Co-first author

Table of contents

Entry	*Subject*	*Page*
1	Table 1.Physical properties of geopolymer and $NiFe_2O_4$/geopolymer samples from BET analysis	S2
2	Table 2. Optimization of different parameters for model reaction	S3
3	Fig. S1. Reusability of nanocatalyst in the synthesis of **4a**	S4
4	Fig. S2. FT-IR spectrums of recycled nanocatalyst	S5
5	Fig. S3. EDX analysis of recycled nanocatalyst	S6
6	Fig. S4. FT-IR spectrum of the product (**4a**)	S7
7	Fig. S5. ^{1}H NMR of the product (**4a**)	S8
8	Fig. S6. ^{13}C NMR of the product (**4a**)	S9
9	Fig. S7. FT-IR spectrum of the product (**4i**)	S10
10	Fig. S8. ^{1}H NMR of the product (**4i**)	S11
11	Fig. S9. ^{13}C NMR of the product **(4i)**	S12

Table 1. Physical properties of geopolymer and $NiFe_2O_4$/geopolymer samples from BET analysis.

Physical Properties	Geopolymer Sample	$NiFe_2O_4$/geopolymer
Surface area (m^2/g)	1.13	0.30
Pore size (nm)	14.28	19.82
The total pore volume of pores (cm^3/g)	0.004	0.069

Table 2. Optimization of different parameters for model reaction.[a]

Entry	Solvent	Catalyst (g)	Condition/Temperature (˚C)	Time (min)	Yield[b] (%)
1	EtOH	Nanocomposite (0.03)	r.t. / 27	277	75
2	EtOH	Nanocomposite (0.03)	Reflux / 90	155	89
3	Free-solvent	Nanocomposite (0.03)	Ultrasonic bath / 80	18	trace
4	H_2O	Nanocomposite (0.03)	Ultrasonic bath / 80	18	No reaction
5	EtOH	Nanocomposite (0.03)	Ultrasonic bath / 80	18	91
6	MeOH	Nanocomposite (0.03)	Ultrasonic bath / 80	18	76
7	CH_2Cl_2	Nanocomposite (0.03)	Ultrasonic bath / 80	18	84
8	CH_3CN	Nanocomposite (0.03)	Ultrasonic bath / 80	18	69
9	Toluene	Nanocomposite (0.03)	Ultrasonic bath / 80	18	trace
10	EtOH	-	Ultrasonic bath / 80	30	trace
11	EtOH	Bentonite (0.03)	Ultrasonic bath / 80	25	No reaction
12	EtOH	$NiFe_2O_4$ (0.03)	Ultrasonic bath / 80	25	38
13	EtOH	Geopolymer (0.03)	Ultrasonic bath / 80	25	20
14	EtOH	Nanocomposite (0.01)	Ultrasonic bath / 80	18	65
15	EtOH	Nanocomposite (0.02)	Ultrasonic bath / 80	18	71
16	EtOH	Nanocomposite (0.04)	Ultrasonic bath / 80	18	89
17	EtOH	Nanocomposite (0.05)	Ultrasonic bath / 80	18	90

[a]The reaction condition: benzil (0.8 mmol), benzaldehyde (0.8 mmol), ammonium acetate (2.0 mmol), Ethanol (7 mL) ultrasonic irradiations.

[b] Isolated yield.

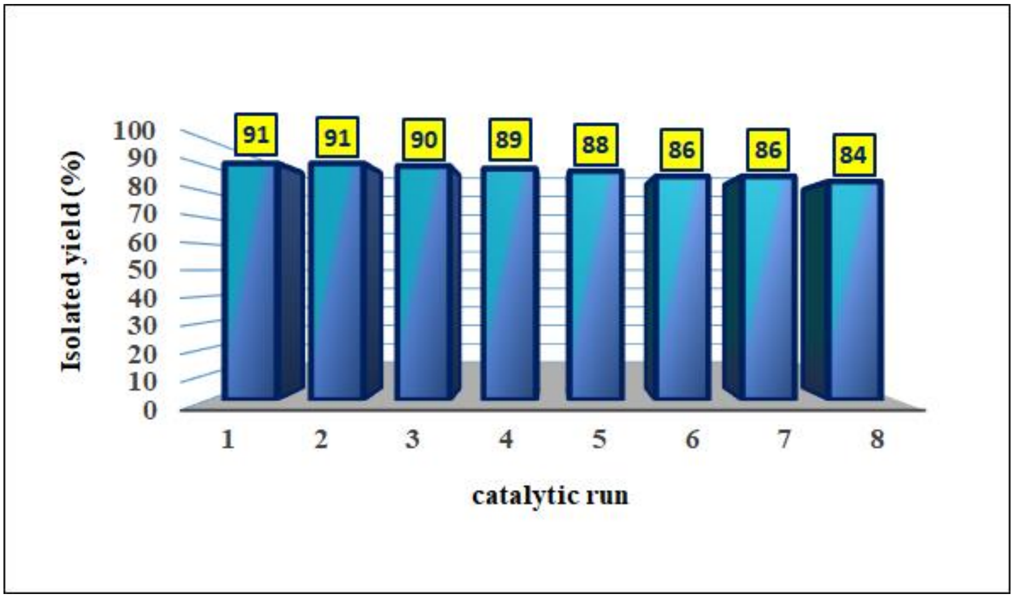

Fig. S1. The Reusability of $NiFe_2O_4$/geopolymer nanocatalyst in the synthesis of **4a.**

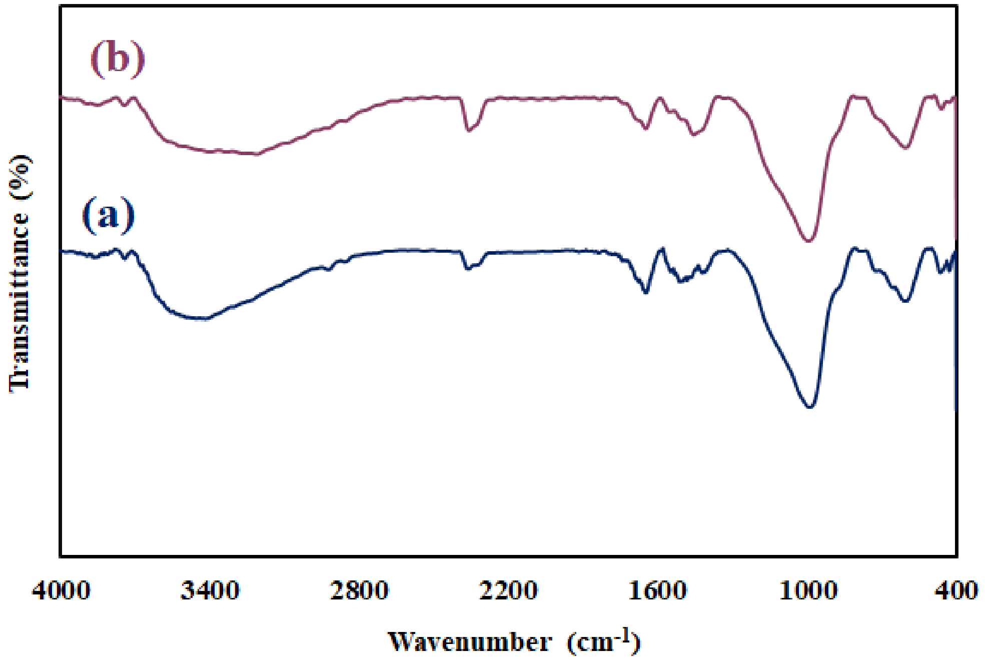

Fig. S2. FT-IR spectra of (a) $NiFe_2O_4$/geopolymer nanocomposite, (b) recycled nanocatalyst.

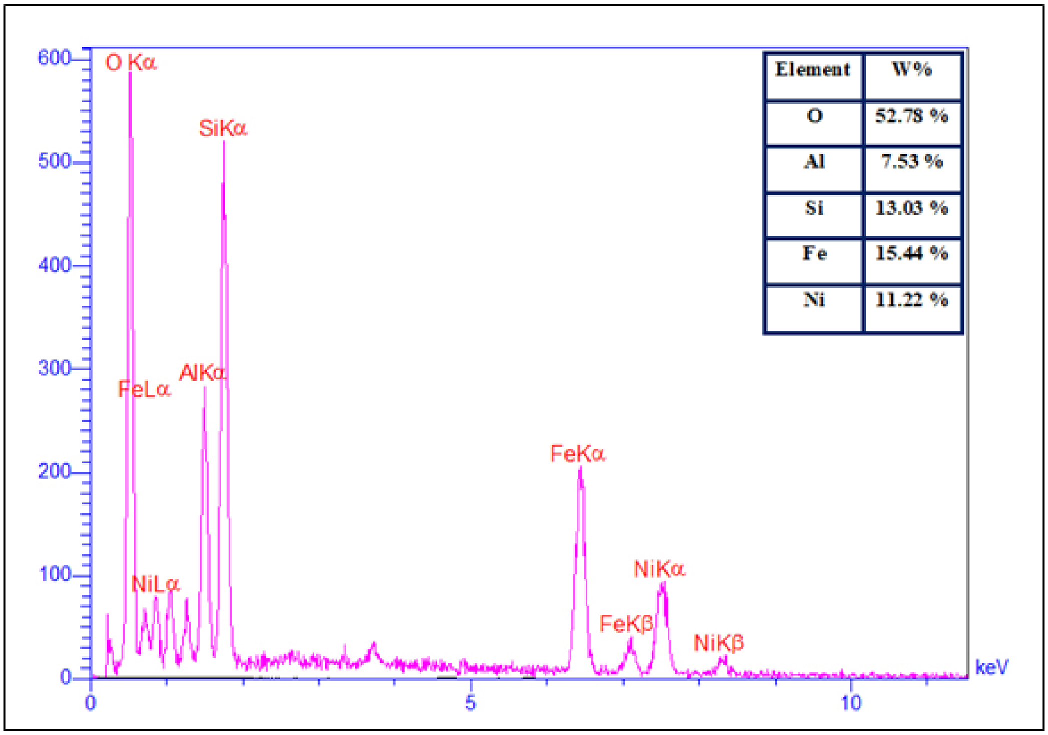

Fig. S3. The EDX analysis of recycled $NiFe_2O_4$/geopolymer nanocomposite.

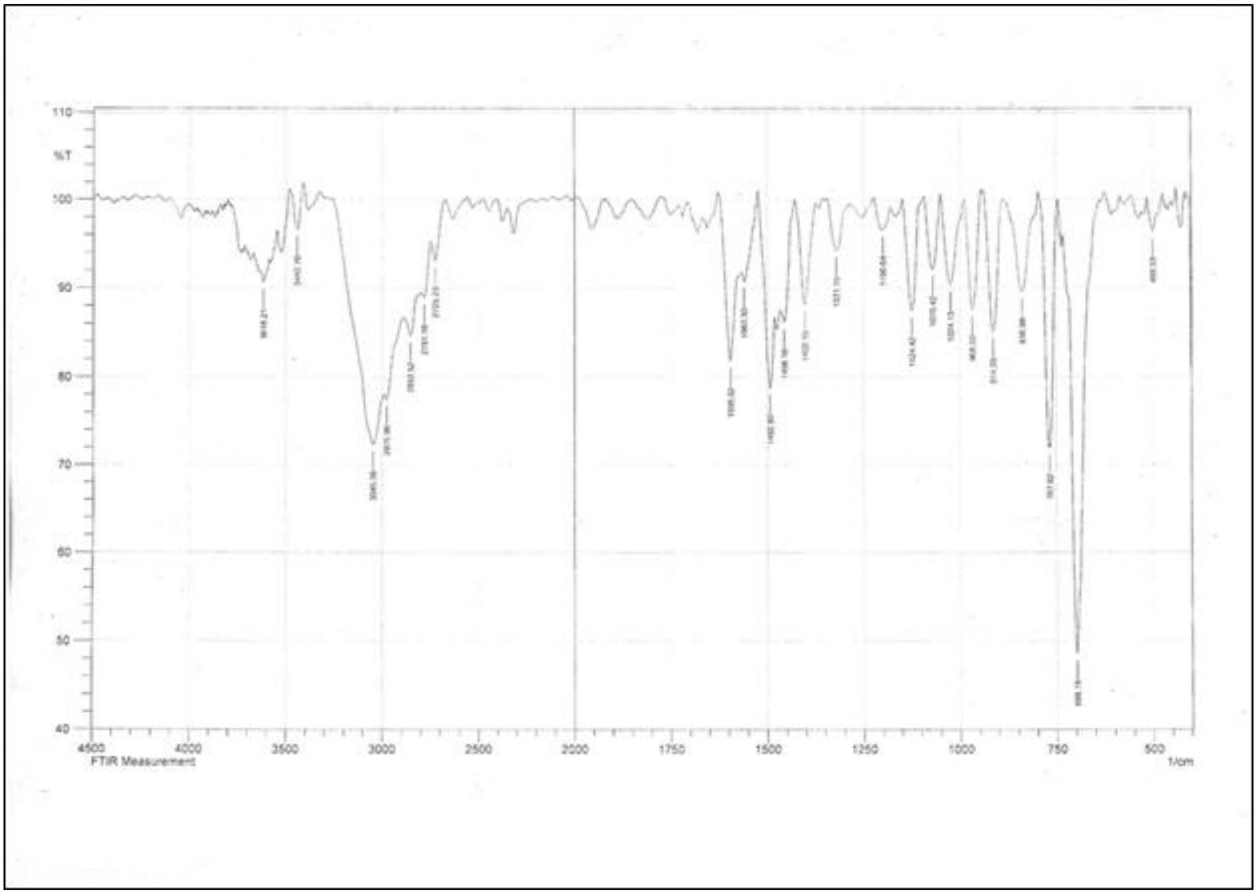

Fig. S4. The FT-IR spectrum of the product (**4a**).

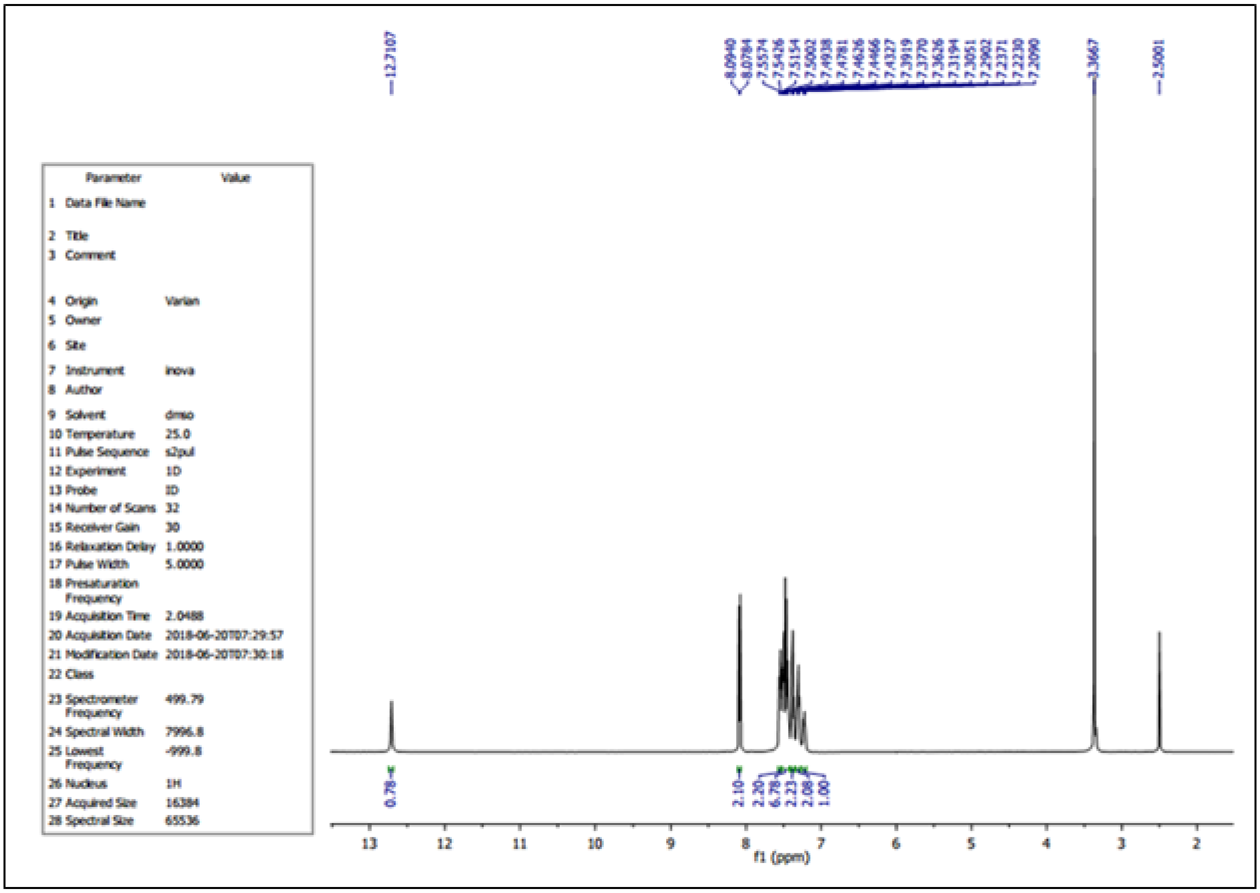

Fig. **S**5**.** The ^{1}H NMR of the product (**4a**).

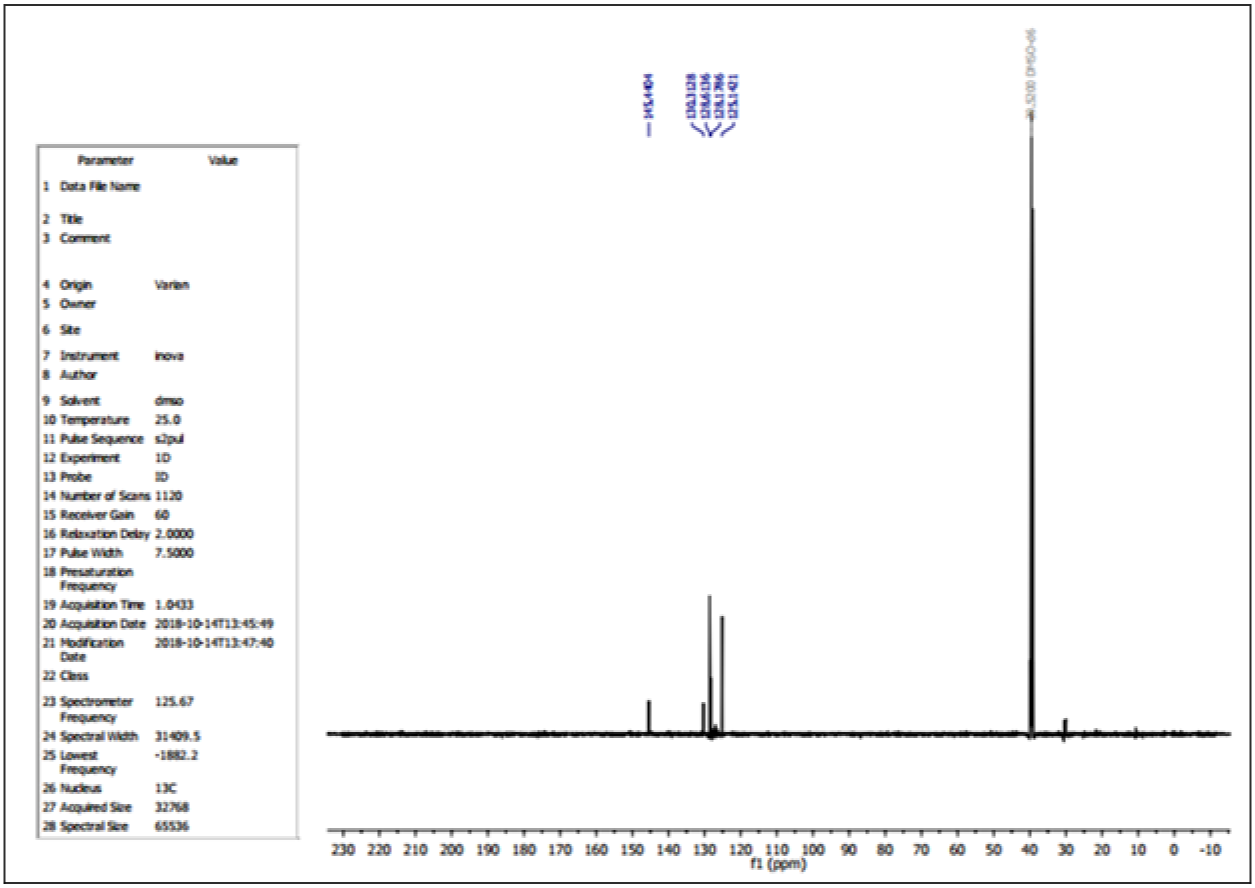

Fig. S6. The ^{13}C NMR of the product (**4a**).

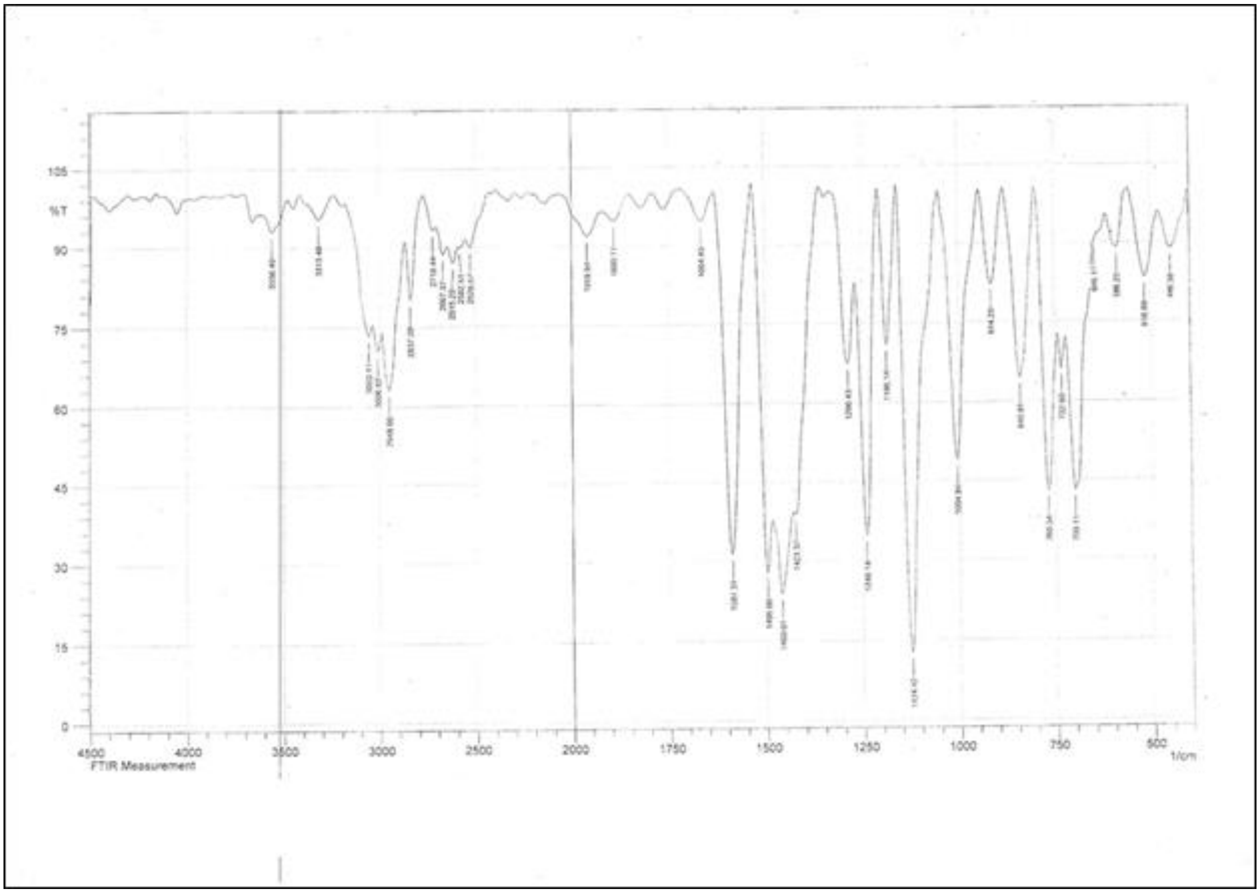

Fig. S7. The FT-IR spectrum of the product (**4i**).

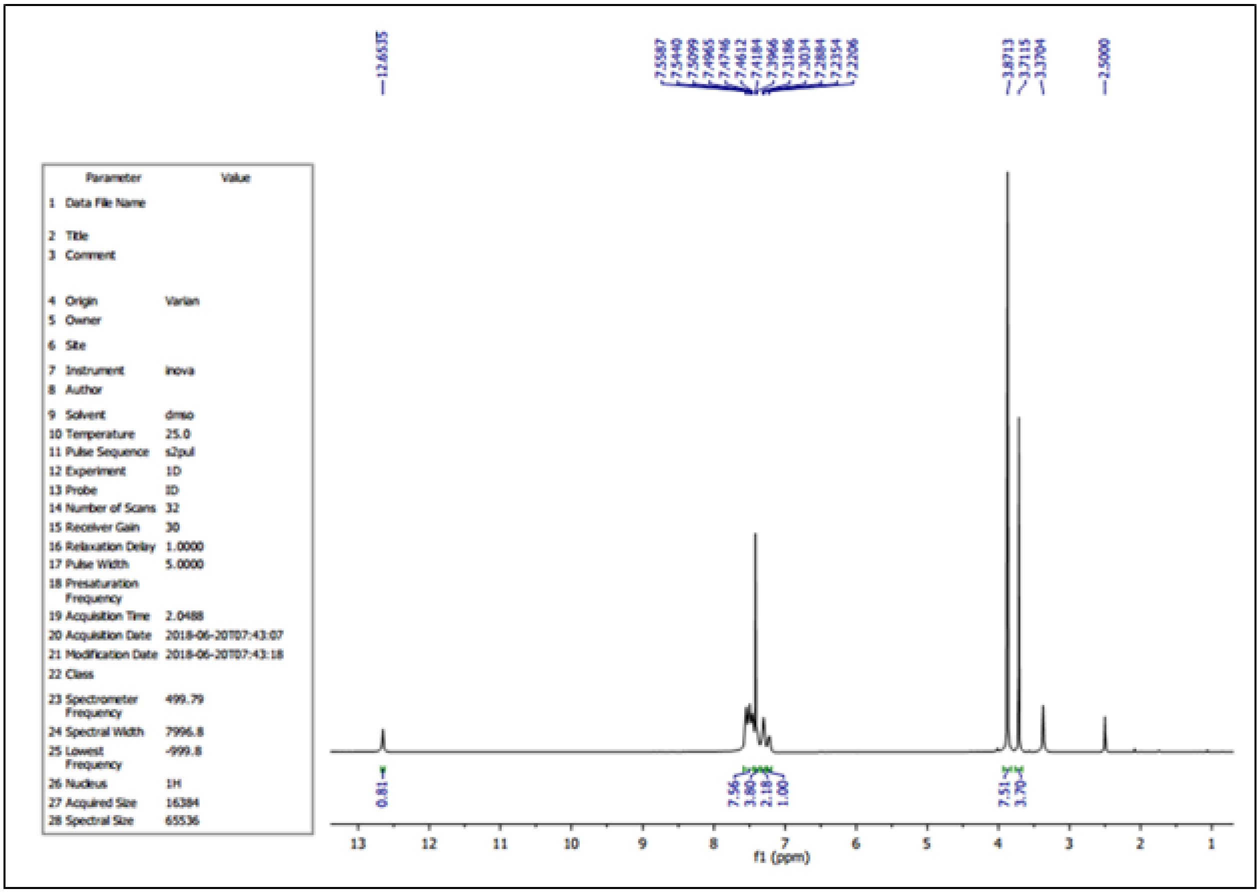

Fig. S8. The ^{1}H NMR of the product (**4i**).

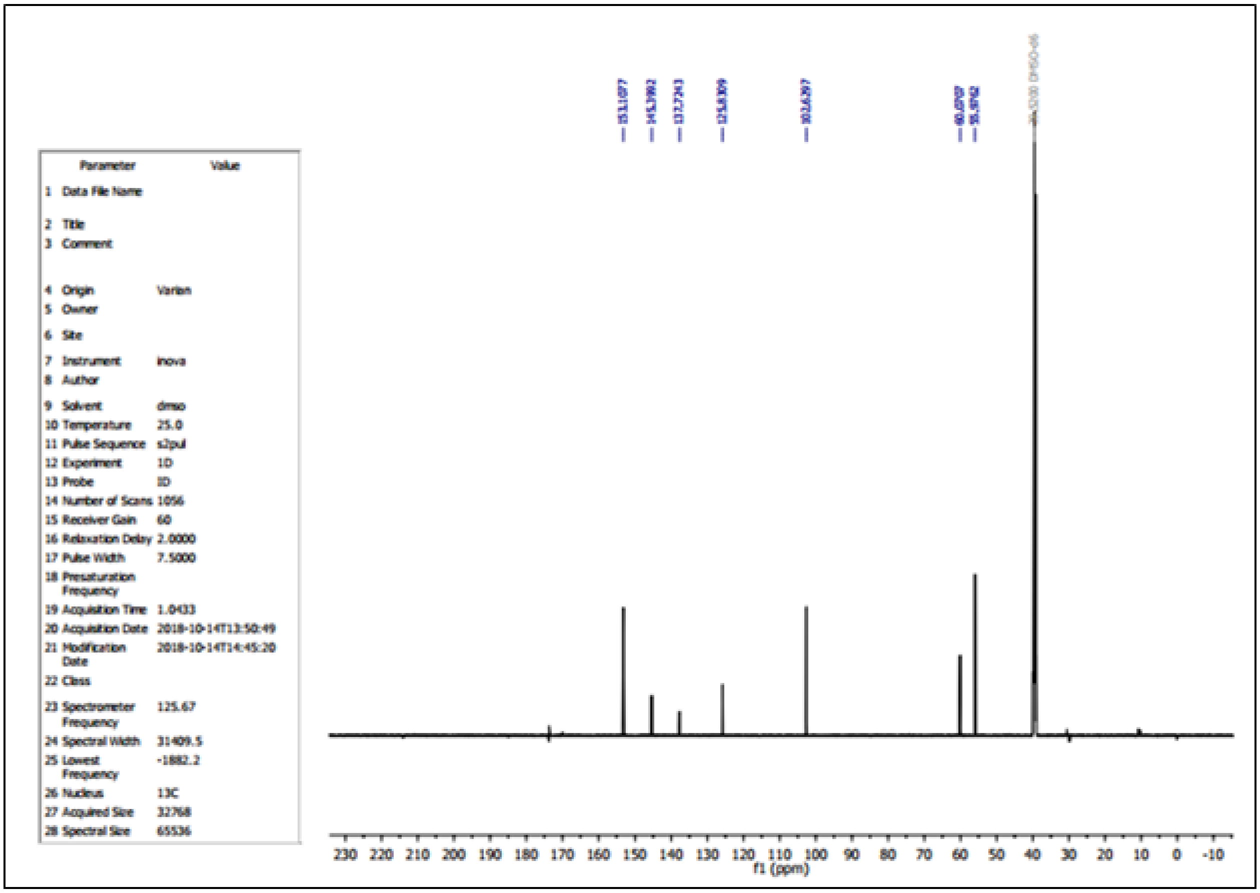

Fig. S9. The ^{13}C NMR of the product (**4i**).
